# 3-Methyl-1,4-dioxo-1,4-dihydro­naphthalen-2-yl 4-amino­benzoate

**DOI:** 10.1107/S1600536808005308

**Published:** 2008-03-14

**Authors:** Massimo Bambagiotti-Alberti, Gianluca Bartolucci, Bruno Bruni, Silvia Coran, Massimo Di Vaira

**Affiliations:** a Dipartimento di Scienze Farmaceutiche Universitá di Firenze Via U. Schiff 6, I-50019 Sesto Fiorentino Firenze, Italy; b Dipartimento di Chimica Universitá di Firenze Via della Lastruccia 3, I-50019 Sesto Fiorentino Firenze, Italy

## Abstract

The crystal structure of the title compound, C_18_H_13_NO_4_, the oxidized form of the drug aminaftone used in venous disease therapy, is characterized by the presence of ribbons of hydrogen-bonded mol­ecules parallel to the [111] crystallographic direction and by stacking inter­actions between rings [centroid–centroid distance between quinone rings = 3.684 (3) Å and between amino­benzoate rings = 4.157 (3) Å] along the ribbons.

## Related literature

For related literature, see: De Anna *et al.* (1989 ▶); Martinez *et al.* (2005 ▶).
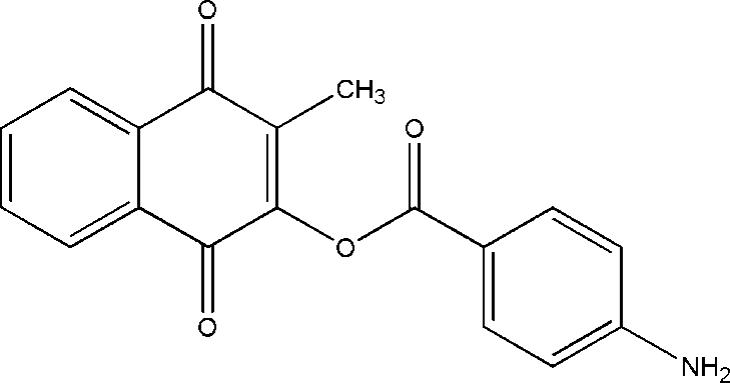


## Experimental

### 

#### Crystal data

C_18_H_13_NO_4_
*M* *_r_* = 307.29
Triclinic, 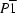

*a* = 7.6217 (6) Å
*b* = 9.6142 (7) Å
*c* = 10.6456 (7) Å
α = 101.618 (6)°
β = 110.770 (7)°
γ = 89.019 (6)°
*V* = 713.18 (10) Å^3^
*Z* = 2
Cu *K*α radiation
μ = 0.85 mm^−1^
*T* = 170 (2) K
0.60 × 0.20 × 0.05 mm

#### Data collection

Oxford Diffraction Xcalibur PX Ultra CCD diffractometer
Absorption correction: multi-scan (*ABSPACK*; Oxford Diffraction, 2006 ▶) *T*_min_ = 0.501, *T*_max_ = 1.000 (expected range = 0.480–0.959)
6339 measured reflections
2453 independent reflections
1845 reflections with *I* > 2σ(*I*)
*R*_int_ = 0.022

#### Refinement

*R*[*F* ^2^ > 2σ(*F* ^2^)] = 0.041
*wR*(*F* ^2^) = 0.142
*S* = 1.12
2453 reflections
215 parameters
H atoms treated by a mixture of independent and constrained refinement
Δρ_max_ = 0.17 e Å^−3^
Δρ_min_ = −0.23 e Å^−3^

### 

Data collection: *CrysAlisPro CCD* (Oxford Diffraction, 2006 ▶); cell refinement: *CrysAlisPro CCD*; data reduction: *CrysAlisPro RED* (Oxford Diffraction, 2006 ▶); program(s) used to solve structure: *SIR97* (Altomare *et al.*, 1999 ▶); program(s) used to refine structure: *SHELXL97* (Sheldrick, 2008 ▶); molecular graphics: *ORTEP-3* (Farrugia, 1997 ▶) and *PLATON* (Spek, 2003 ▶); software used to prepare material for publication: *SHELXL97* and *PARST* (Nardelli, 1995 ▶).

## Supplementary Material

Crystal structure: contains datablocks global, I. DOI: 10.1107/S1600536808005308/rk2078sup1.cif

Structure factors: contains datablocks I. DOI: 10.1107/S1600536808005308/rk2078Isup2.hkl

Additional supplementary materials:  crystallographic information; 3D view; checkCIF report

## Figures and Tables

**Table 1:** Hydrogen-bond geometry (Å, °)

*D*—H⋯*A*	*D*—H	H⋯*A*	*D*⋯*A*	*D*—H⋯*A*
N—H2*N*⋯O3^i^	0.95 (3)	2.13 (3)	2.960 (2)	145.6 (19)
N—H1*N*⋯O4^ii^	0.89 (2)	2.25 (2)	3.045 (2)	147.3 (19)

Symmetry codes: (i) 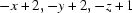; (ii) 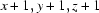.

**Fig. 1.**
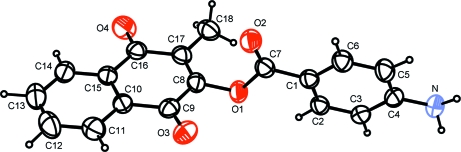
A view of the molecule of I with the atom numbering schene. Displacement ellipsoids are drawn at the 50% probability level. H atoms are presented as a small spheres of arbitrary radius.

**Fig. 2.**
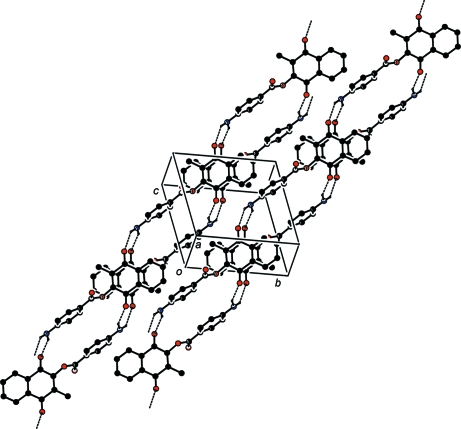
A view of the crystal packing in the structure of I, showing the presence of ribbons formed by H–bonded molecules. H–bonds are denoted by dashed lines.

## supplementary crystallographic information

### Comment

Previuos studies concerning the quality control of aminaftone, 4–aminobenzoic acid 1,4–dihydroxy–3–methyl–naphtalen–2–yl ester, which is the active pharmaceutical ingredient of some commercial drugs used in the therapy of chronic venous and lymphatic stasis (De Anna *et al.*, 1989; Martinez *et al.*, 2005), have shown that this drug substance rapidly undergoes oxidation in solution at room temperature. The oxidized compound, featuring a quinone ring instead of a hydroquinone moiety, is also a potential impurity of the bulk drug. A structure determination of the oxidized form, I, has been undertaken at 170 K.

There are two molecules of I, related by the inversion centre, in the triclinic unit cell. The molecular geometry and labelling are shown in Fig 1. Bond distances are consistent with the presence of the quinonic form. Each molecule behaves as a hydrogen–bond donor, toward two other molecules, through its aminic H atoms: N—H2N···O3^i^ (N···O3^i^ = 2.960 (2) Å, N—H2N···O3^i^ = 146 (2)°; symmetry code (i): 2 - *x*, 2 - *y*, 1 - *z*) and N—H1N···O4^ii^ (N···O4^ii^ = 3.045 (2) Å, N—H1N···O4^ii^ = 147 (2)°; symmetry code (ii): 1 + *x*, 1 + *y*, 1 + *z*). Conversely, through the above hydrogen bonds each molecule behaves as an acceptor from two separate molecules, by means of its quinone O atoms. As a result, ribbons of hydrogen–bonded molecules, parallel to the crystallographic [1 1 1] direction, are formed (Fig. 2). Moreover, stacking interactions occur along the ribbons, with 3.684 (3)Å distance between the centroids of symmetry–related quinone rings and 4.157 (3)Å distance between the centroids of the aminobenzoic rings, the shortest C···C contact distances between atoms of facing rings being C8···C16^iii^ = 3.532 (3)Å ((iii): 1 - *x*, 1 - *y*, -*z*) and C2···C3^i^ = 3.286 (2) Å, respectively, for the above two types of interactions. The largest deviation (0.098 (1) Å) from the plane of the aminobenzoic group is presented by the carbonylic O2 atom whereas, among the atoms lying on the naphtoquinone plane, the hinge atom O1 exhibits the largest deviation (0.141 (2) Å) from that plane; the angle between these two planes measures 84.46 (3)°.

### Experimental

Samples of aminonaftone were kindly provided by SIMS (SIMS srl, Reggello Firenze, Italy). Crystals of I, suitable for *X*–ray diffraction analysis, were obtained by slow evaporation from methanol solutions of aminaftone.

### Refinement

Crystals did not diffract strongly and it was deemed that collecting data at θ higher than 72° would not yield improvement. Hydrogen atoms were in geometrically generated positions, riding, except for the amino H atoms, which were refined freely. The constraint *U(H)* = 1.2*U*_eq_(C,*N*), or 1.5*U*_eq_(C) for methyl group H atoms, was applied. Range of bond distances involving refined hydrogen atoms: N—H 0.89–0.95 Å.

### Crystal data

**Table:**

C_18_H_13_NO_4_	*Z* = 2
*M**_r_* = 307.29	*F*_000_ = 320
Triclinic, *P*1	*D*_x_ = 1.431 Mg m^−^^3^
Hall symbol: -P 1	Cu *K*α radiation λ = 1.54180 Å
*a* = 7.6217 (6) Å	Cell parameters from 3411 reflections
*b* = 9.6142 (7) Å	θ = 9.6–53.4º
*c* = 10.6456 (7) Å	µ = 0.85 mm^−^^1^
α = 101.618 (6)º	*T* = 170 (2) K
β = 110.770 (7)º	Flat prism, red
γ = 89.019 (6)º	0.60 × 0.20 × 0.05 mm
*V* = 713.18 (10) Å^3^	

### Data collection

**Table:**

Oxford Diffraction Xcalibur PX Ultra CCD diffractometer	2453 independent reflections
Radiation source: Fine–focus sealed tube	1845 reflections with *I* > 2σ(*I*)
Monochromator: Oxford Diffraction Enhance ULTRA assembly	*R*_int_ = 0.022
Detector resolution: 8.1241 pixels mm^-1^	θ_max_ = 72.4º
*T* = 170(2) K	θ_min_ = 4.5º
ω scans	*h* = −9→9
Absorption correction: multi-scan(ABSPACK; Oxford Diffraction, 2006)	*k* = −11→11
*T*_min_ = 0.501, *T*_max_ = 1.000	*l* = −12→12
6339 measured reflections	

### Refinement

**Table:**

Refinement on *F*^2^	Secondary atom site location: Difmap
Least-squares matrix: Full	Hydrogen site location: Difmap
*R*[*F*^2^ > 2σ(*F*^2^)] = 0.041	H atoms treated by a mixture of independent and constrained refinement
*wR*(*F*^2^) = 0.142	*w* = 1/[σ^2^(*F*_o_^2^) + (0.0941*P*)^2^ + 0.0064*P*] where *P* = (*F*_o_^2^ + 2*F*_c_^2^)/3
*S* = 1.12	(Δ/σ)_max_ < 0.001
2453 reflections	Δρ_max_ = 0.17 e Å^−^^3^
215 parameters	Δρ_min_ = −0.23 e Å^−^^3^
Primary atom site location: Direct	Extinction correction: None

### Special details

**Table:**

Geometry. All s.u.'s (except the s.u. in the dihedral angle between two l.s. planes) are estimated using the full covariance matrix. The cell s.u.'s are taken into account individually in the estimation of s.u.'s in distances, angles and torsion angles; correlations between s.u.'s in cell parameters are only used when they are defined by crystal symmetry. An approximate (isotropic) treatment of cell s.u.'s is used for estimating s.u.'s involving l.s. planes.
Refinement. Refinement of *F*^2^ against ALL reflections. The weighted *R*–factor w*R* and goodness of fit *S* are based on *F*^2^, conventional *R*–factors *R* are based on *F*, with *F* set to zero for negative *F*^2^. The threshold expression of *F*^2^ > 2σ(*F*^2^) is used only for calculating *R*–factors(gt) *etc*. and is not relevant to the choice of reflections for refinement. *R*–factors based on *F*^2^ are statistically about twice as large as those based on *F*, and *R*–factors based on ALL data will be even larger.

### Fractional atomic coordinates and isotropic or equivalent isotropic displacement parameters (Å^2^)

**Table:**

	*x*	*y*	*z*	*U*_iso_*/*U*_eq_	
C1	0.6680 (2)	1.03157 (16)	0.34314 (16)	0.0361 (4)	
C2	0.8411 (2)	1.01124 (16)	0.32984 (16)	0.0373 (4)	
H2	0.8613	0.9234	0.2787	0.045*	
C3	0.9857 (3)	1.11583 (17)	0.38923 (16)	0.0392 (4)	
H3	1.1038	1.0996	0.3791	0.047*	
C4	0.9568 (3)	1.24645 (17)	0.46471 (16)	0.0398 (4)	
N	1.0987 (3)	1.35138 (17)	0.52199 (17)	0.0508 (4)	
H1N	1.083 (3)	1.425 (3)	0.582 (2)	0.061*	
H2N	1.225 (4)	1.326 (2)	0.542 (2)	0.061*	
C5	0.7833 (3)	1.26644 (17)	0.47801 (17)	0.0449 (5)	
H5	0.7624	1.3542	0.5289	0.054*	
C6	0.6405 (3)	1.16127 (18)	0.41876 (17)	0.0431 (4)	
H6	0.5226	1.1770	0.4294	0.052*	
C7	0.5122 (3)	0.92331 (17)	0.27898 (16)	0.0384 (4)	
O1	0.57285 (17)	0.79775 (12)	0.22176 (13)	0.0464 (4)	
O2	0.35157 (19)	0.93363 (13)	0.27039 (14)	0.0498 (4)	
C8	0.4401 (2)	0.68767 (16)	0.14533 (17)	0.0377 (4)	
C9	0.4125 (2)	0.58405 (18)	0.22396 (17)	0.0386 (4)	
O3	0.48719 (18)	0.60587 (14)	0.34797 (12)	0.0497 (4)	
C10	0.2922 (2)	0.45428 (17)	0.14241 (18)	0.0388 (4)	
C11	0.2663 (3)	0.3494 (2)	0.2090 (2)	0.0498 (5)	
H11	0.3227	0.3623	0.3060	0.060*	
C12	0.1572 (3)	0.2263 (2)	0.1317 (2)	0.0602 (6)	
H12	0.1414	0.1536	0.1761	0.072*	
C13	0.0713 (3)	0.2085 (2)	−0.0091 (2)	0.0552 (5)	
H13	−0.0036	0.1239	−0.0607	0.066*	
C14	0.0936 (3)	0.31287 (18)	−0.0752 (2)	0.0466 (5)	
H14	0.0330	0.3008	−0.1719	0.056*	
C15	0.2056 (2)	0.43605 (16)	0.00057 (17)	0.0376 (4)	
O4	0.1557 (2)	0.53290 (14)	−0.19534 (13)	0.0593 (4)	
C16	0.2311 (2)	0.54714 (17)	−0.07165 (17)	0.0405 (4)	
C17	0.3535 (2)	0.67684 (17)	0.01047 (18)	0.0393 (4)	
C18	0.3757 (3)	0.7879 (2)	−0.0642 (2)	0.0516 (5)	
H181	0.4980	0.8401	−0.0144	0.077*	
H182	0.2753	0.8541	−0.0699	0.077*	
H183	0.3683	0.7421	−0.1570	0.077*	

### Atomic displacement parameters (Å^2^)

**Table:**

	*U*^11^	*U*^22^	*U*^33^	*U*^12^	*U*^13^	*U*^23^
C1	0.0393 (10)	0.0345 (8)	0.0337 (8)	0.0027 (8)	0.0114 (7)	0.0083 (6)
C2	0.0443 (10)	0.0325 (7)	0.0329 (8)	0.0035 (8)	0.0118 (7)	0.0059 (6)
C3	0.0385 (10)	0.0398 (8)	0.0357 (8)	0.0033 (8)	0.0088 (7)	0.0085 (7)
C4	0.0435 (11)	0.0382 (8)	0.0318 (8)	−0.0001 (8)	0.0065 (7)	0.0066 (6)
N	0.0484 (10)	0.0413 (8)	0.0487 (9)	−0.0045 (8)	0.0063 (7)	−0.0016 (7)
C5	0.0529 (12)	0.0360 (8)	0.0417 (10)	0.0019 (9)	0.0175 (8)	−0.0017 (7)
C6	0.0449 (11)	0.0407 (9)	0.0436 (9)	0.0035 (8)	0.0176 (8)	0.0053 (7)
C7	0.0443 (11)	0.0347 (8)	0.0362 (8)	0.0032 (8)	0.0136 (7)	0.0091 (6)
O1	0.0370 (7)	0.0339 (6)	0.0615 (8)	−0.0006 (5)	0.0156 (6)	−0.0013 (5)
O2	0.0410 (8)	0.0444 (7)	0.0622 (8)	0.0016 (6)	0.0202 (6)	0.0040 (6)
C8	0.0326 (9)	0.0313 (7)	0.0480 (10)	0.0026 (7)	0.0164 (7)	0.0021 (7)
C9	0.0322 (9)	0.0423 (8)	0.0402 (9)	0.0065 (8)	0.0137 (7)	0.0050 (7)
O3	0.0438 (8)	0.0612 (8)	0.0397 (7)	0.0022 (6)	0.0124 (5)	0.0057 (6)
C10	0.0331 (9)	0.0366 (8)	0.0476 (10)	0.0031 (8)	0.0153 (7)	0.0092 (7)
C11	0.0484 (12)	0.0500 (10)	0.0583 (11)	0.0063 (9)	0.0248 (9)	0.0173 (9)
C12	0.0629 (14)	0.0438 (10)	0.0877 (16)	−0.0002 (10)	0.0405 (12)	0.0192 (10)
C13	0.0464 (12)	0.0389 (9)	0.0817 (15)	−0.0021 (9)	0.0316 (10)	−0.0003 (9)
C14	0.0366 (10)	0.0391 (9)	0.0578 (11)	0.0037 (8)	0.0157 (8)	−0.0017 (8)
C15	0.0320 (9)	0.0330 (8)	0.0472 (10)	0.0068 (7)	0.0168 (7)	0.0032 (7)
O4	0.0774 (11)	0.0530 (7)	0.0385 (7)	0.0067 (7)	0.0124 (6)	0.0053 (6)
C16	0.0386 (10)	0.0385 (8)	0.0414 (10)	0.0089 (8)	0.0121 (7)	0.0064 (7)
C17	0.0368 (10)	0.0355 (8)	0.0477 (10)	0.0073 (8)	0.0173 (8)	0.0095 (7)
C18	0.0535 (12)	0.0465 (10)	0.0626 (12)	0.0078 (9)	0.0245 (9)	0.0222 (9)

### Geometric parameters (Å, °)

**Table:**

C1—C2	1.383 (3)	C9—O3	1.213 (2)
C1—C6	1.398 (2)	C9—C10	1.480 (2)
C1—C7	1.463 (2)	C10—C15	1.392 (2)
C2—C3	1.383 (2)	C10—C11	1.397 (2)
C2—H2	0.9500	C11—C12	1.387 (3)
C3—C4	1.407 (2)	C11—H11	0.9500
C3—H3	0.9500	C12—C13	1.382 (3)
C4—N	1.371 (2)	C12—H12	0.9500
C4—C5	1.385 (3)	C13—C14	1.381 (3)
N—H1N	0.89 (2)	C13—H13	0.9500
N—H2N	0.95 (3)	C14—C15	1.394 (2)
C5—C6	1.378 (2)	C14—H14	0.9500
C5—H5	0.9500	C15—C16	1.488 (2)
C6—H6	0.9500	O4—C16	1.215 (2)
C7—O2	1.199 (2)	C16—C17	1.487 (2)
C7—O1	1.388 (2)	C17—C18	1.498 (2)
O1—C8	1.3827 (19)	C18—H181	0.9800
C8—C17	1.333 (2)	C18—H182	0.9800
C8—C9	1.485 (2)	C18—H183	0.9800
			
C2—C1—C6	118.63 (15)	C15—C10—C11	120.01 (16)
C2—C1—C7	122.37 (15)	C15—C10—C9	120.57 (14)
C6—C1—C7	118.99 (16)	C11—C10—C9	119.42 (16)
C1—C2—C3	121.52 (15)	C12—C11—C10	119.25 (19)
C1—C2—H2	119.2	C12—C11—H11	120.4
C3—C2—H2	119.2	C10—C11—H11	120.4
C2—C3—C4	119.58 (17)	C13—C12—C11	120.62 (17)
C2—C3—H3	120.2	C13—C12—H12	119.7
C4—C3—H3	120.2	C11—C12—H12	119.7
N—C4—C5	121.40 (16)	C14—C13—C12	120.40 (17)
N—C4—C3	119.83 (18)	C14—C13—H13	119.8
C5—C4—C3	118.76 (15)	C12—C13—H13	119.8
C4—N—H1N	116.7 (15)	C13—C14—C15	119.68 (18)
C4—N—H2N	119.0 (13)	C13—C14—H14	120.2
H1N—N—H2N	114 (2)	C15—C14—H14	120.2
C6—C5—C4	121.14 (16)	C10—C15—C14	120.02 (16)
C6—C5—H5	119.4	C10—C15—C16	120.62 (14)
C4—C5—H5	119.4	C14—C15—C16	119.37 (16)
C5—C6—C1	120.36 (17)	O4—C16—C17	120.01 (15)
C5—C6—H6	119.8	O4—C16—C15	121.32 (15)
C1—C6—H6	119.8	C17—C16—C15	118.67 (14)
O2—C7—O1	121.27 (15)	C8—C17—C16	118.88 (14)
O2—C7—C1	128.52 (16)	C8—C17—C18	123.35 (16)
O1—C7—C1	110.20 (15)	C16—C17—C18	117.75 (15)
C8—O1—C7	118.14 (13)	C17—C18—H181	109.5
C17—C8—O1	120.33 (14)	C17—C18—H182	109.5
C17—C8—C9	124.77 (14)	H181—C18—H182	109.5
O1—C8—C9	114.83 (14)	C17—C18—H183	109.5
O3—C9—C10	122.82 (15)	H181—C18—H183	109.5
O3—C9—C8	120.89 (15)	H182—C18—H183	109.5
C10—C9—C8	116.28 (14)		
			
C6—C1—C2—C3	−0.1 (2)	C8—C9—C10—C11	177.37 (17)
C7—C1—C2—C3	178.82 (14)	C15—C10—C11—C12	1.4 (3)
C1—C2—C3—C4	−0.2 (2)	C9—C10—C11—C12	−178.40 (17)
C2—C3—C4—N	−178.97 (15)	C10—C11—C12—C13	−1.5 (3)
C2—C3—C4—C5	0.3 (2)	C11—C12—C13—C14	0.3 (3)
N—C4—C5—C6	179.17 (15)	C12—C13—C14—C15	0.8 (3)
C3—C4—C5—C6	−0.1 (3)	C11—C10—C15—C14	−0.3 (3)
C4—C5—C6—C1	−0.2 (3)	C9—C10—C15—C14	179.56 (16)
C2—C1—C6—C5	0.3 (2)	C11—C10—C15—C16	179.70 (16)
C7—C1—C6—C5	−178.65 (15)	C9—C10—C15—C16	−0.5 (3)
C2—C1—C7—O2	−171.14 (16)	C13—C14—C15—C10	−0.9 (3)
C6—C1—C7—O2	7.8 (3)	C13—C14—C15—C16	179.15 (17)
C2—C1—C7—O1	7.5 (2)	C10—C15—C16—O4	−179.69 (17)
C6—C1—C7—O1	−173.56 (14)	C14—C15—C16—O4	0.3 (3)
O2—C7—O1—C8	4.8 (2)	C10—C15—C16—C17	1.0 (3)
C1—C7—O1—C8	−174.01 (13)	C14—C15—C16—C17	−179.01 (16)
C7—O1—C8—C17	89.9 (2)	O1—C8—C17—C16	171.84 (14)
C7—O1—C8—C9	−92.86 (17)	C9—C8—C17—C16	−5.1 (3)
C17—C8—C9—O3	−175.24 (17)	O1—C8—C17—C18	−7.0 (3)
O1—C8—C9—O3	7.7 (2)	C9—C8—C17—C18	176.11 (16)
C17—C8—C9—C10	5.5 (3)	O4—C16—C17—C8	−177.60 (17)
O1—C8—C9—C10	−171.56 (14)	C15—C16—C17—C8	1.7 (2)
O3—C9—C10—C15	178.30 (17)	O4—C16—C17—C18	1.3 (3)
C8—C9—C10—C15	−2.5 (2)	C15—C16—C17—C18	−179.42 (16)
O3—C9—C10—C11	−1.9 (3)		

### Hydrogen-bond geometry (Å, °)

**Table:**

*D*—H···*A*	*D*—H	H···*A*	*D*···*A*	*D*—H···*A*
N—H2N···O3^i^	0.95 (3)	2.13 (3)	2.960 (2)	145.6 (19)
N—H1N···O4^ii^	0.89 (2)	2.25 (2)	3.045 (2)	147.3 (19)

Symmetry codes: (i) −*x*+2, −*y*+2, −*z*+1; (ii) *x*+1, *y*+1, *z*+1.
